# Antileukemia Effect of Ciclopirox Olamine Is Mediated by Downregulation of Intracellular Ferritin and Inhibition β-Catenin-c-Myc Signaling Pathway in Glucocorticoid Resistant T-ALL Cell Lines

**DOI:** 10.1371/journal.pone.0161509

**Published:** 2016-08-23

**Authors:** Jianrong Wu, Huajun Liu, Ge Zhang, Ling Gu, Yanle Zhang, Ju Gao, Yuquan Wei, Zhigui Ma

**Affiliations:** 1 Department of Pediatrics, Key Laboratory of Birth Defects and Related Diseases of Women and Children, West China Second University Hospital, Sichuan University, Chengdu, 60041, PR China; 2 State Key Laboratory of Biotherapy and Cancer Center/Collaborative Innovation Center for Biotherapy, West China Hospital, Sichuan University, Chengdu, 610041, PR China; Emory University, UNITED STATES

## Abstract

Ciclopirox olamine (CPX) is an antifungal drug that has been reported to have antitumor effects. In this study we investigated the antileukemia effects and the possible mechanisms of CPX on glucocorticoid (GC)-resistant T-cell acute lymphoblastic leukemia (T-ALL) cell lines. The results indicated that CPX inhibited the growth of GC-resistant T-ALL cells in a time- and dose-dependent manner, and this effect was closely correlated with the downregulation of intracellular ferritin. CPX induced cell cycle arrest at G1 phase by upregulation of cyclin-dependent kinase (CDK) inhibitor of p21 and downregulation of the expressions of cyclin D, retinoblastoma protein (Rb), and phosphorylated Rb (pRb). CPX also enhanced apoptotic cell death by downregulation of anti-apoptotic proteins such as Bcl-2, Bcl-xL_,_ and Mcl-1. More importantly, CPX demonstrated a strong synergistic antileukemia effect with GC and this effect was mediated, at least in part, by inhibition of the β-catenin-c-Myc signaling pathway. These findings suggest that CPX could be a promising antileukemia drug, and modulation of the intracellular ferritin expression might be an effective method in the treatment of ALL. Therefore, integrating CPX into the current GC-containing ALL protocols could lead to the improvement of the outcome of ALL, especially GC-resistant ALL.

## Introduction

Acute lymphoblastic leukemia (ALL) is the most common cancer diagnosed in children. With precise risk-based stratification and optimized risk-directed therapy, it has an overall survival rate of approximately 80%, with certain subsets experiencing a greater than 98% cure rate [1, 2]. Glucocorticoids (GCs), such as prednisolone and dexamethasone (DEX), have been the keystone in the treatment of children with ALL for over 50 years [3, 4]. The initial response to GC therapy has a strong prognostic value in ALL [5–7]. High sensitivity of leukemic blasts to GC determined by 3-(4, 5-dimethylthiazol-2-yl)-2, 5-diphenyltetrazolium bromide (MTT) assay in vitro was also connected with a good prognosis [8]. However, clinical GC resistance occurs in 10–30% of newly-diagnosed ALL patients and is more frequently seen in those with T-lineage ALL (T-ALL), and it always leads to the failure of chemotherapy [9, 10]. T-ALL is a highly malignant tumor representing 10–15% of pediatric and 25% of adult ALL and is clinically regarded as a high risk disease with a relapse rate of about 30% in children [11, 12]. Therefore, defining the molecular mechanism and especially finding a way to overcome GC resistance would contribute to the improvement of the outcome of T-ALL patients.

Ciclopirox olamine (CPX), an antifungal agent commonly used for the dermatologic treatment of mycoses in clinical practice for more than 30 years [13, 14], has been shown recently to have antitumor properties in multiple cancers [15], including hematological malignancies, such as acute leukemia and multiple myeloma [16–19]. Yet, there is rare report on CPX’s antileukemia effect on ALL, especially on ALL with GC resistance. In this study, we used the GC resistant T-ALL cell lines to investigate this antileukemia effect and explore the possible mechanisms of CPX on GC-resistant cell lines. Our findings suggest that CPX might be an attractive new therapeutic approach for GC-resistant T-ALL patients.

## Materials and Methods

### Cell lines

Five T-ALL cell lines were used in the experiments. Jurkat (GC resistant) and Molt-4 (GC resistant) were kindly provided by Dr. Stephan W. Morris (St. Jude Children’s Research Hospital, Memphis, TN), CEM-C7 (GC sensitive) and CEM-C1 (GC resistant) were kindly provided by Dr. E. Brad Thompson (University of Texas Medical Branch), and KE-37 (GC sensitive) was obtained from DSMZ. All cell lines were cultured in RPMI 1640 (HyClone, Thermo SCIENTIFIC), supplemented with 10% newborn bovine serum (NBS, Minhai, Lanzhou, China), 2 mM L-glutamine (Gibco, Carlsbad, CA, USA) at 37°C in a humidified 5% CO_2_ in-air atmosphere.

### Reagents and antibodies

CPX (Sigma, St. Louis, MO, USA) was dissolved in ethanol to make the concentration of the stock solution of 1x10^5^ *μ*M. The final concentration of ethanol in the medium was no more than 0.02%, at which cell proliferation/growth or viability was not obviously altered. 3-(4,5-dimethylthiazol-2-yl)-2,5-diphenyltetrazolium bromide (MTT) and propidium iodide (PI) were purchased from Sigma. The Annexin V-PI kit was purchased from Keygen (Nanjing, China). Enhanced chemiluminescence (ECL) was purchased from Pierce (Rockford, IL, USA). Antibodies to ferritin, Bim, Mcl-1, cyclins A and D, Caspase-3, Rb, c-Myc, phospho-Rb, secondary antibodies of HRP-conjugated donkey anti-rabbit, and sheep anti-mouse were obtained from Cell Signaling Technology (Beverly, MA, USA). The antibody to p21 was purchased from BD Bioscience (San Jose, CA, USA), the antibody to β-catein was obtained from Millipore (Billerica, Massachusetts, USA), and the anti-GAPDH antibody was obtained from Kangchen Bio-Tech (Shanghai, China).

### Cell treatment

Logarithmically growing cells were harvested and replaced in 96- or 6-well or 10mm sterile plastic culture plates, and different concentrations of CPX (1, 1.25, 2.5, 5, 10, 20, and 40 *μ*M) and 0.02% ethanol (control) were added respectively. At the end of the incubation period, cells were transferred to sterile centrifuge tubes, pelleted by centrifugation at 400 x g at room temperature for 5 min, and prepared for analysis as described below.

### Proliferation assay

Logarithmically growing cells were seeded in 96-well plates (20,000 cells per well), and 0.5 mg/mL MTT (final concentration) was added to each well, and the plates were incubated for 4 h at 37°C. Then, 100% (v/v) of a solubilization solution (10% SDS in 0.01 M HCl) was added to each well, and the plates were reincubated for 24 h at 37°C. Spectrophotometric absorbance was measured at 570 nm (reference 690 nm) using a multi-plate reader (Multiskan Spectrum, Thermo Electron Co., Vantaa, Finland). Values were obtained by comparing these cells with their respective controls.

### Cell cycle analysis

For each analysis, 10^6^ cells were harvested after treatment, then washed by phosphate-buffered saline (PBS, pH 7.36) at 900 x g for 3 min twice and fixed overnight in 70% ethanol at 4°C. Cells were then washed and stained with 5 *μ*g/ml PI in the presence of DNAse free RNAse (Sigma). After 30 min at 4°C, the cells were analyzed using Cytomics FC 500 (Beckman-Coulter, Fullerton, CA).

### Assay for apoptosis

The samples were washed with PBS twice and re-suspended in 500 *μ*l of binding buffer containing 5 *μ*l of Annexin V-FITC stock solution and 5 *μ*l of PI for determination of phosphatidyl serine exposure on the outer plasma membrane. After incubation for 10 min at room temperature in a light-protected area, the samples were quantified by flow cytometry with Cytomics FC 500.

### Western blot analysis

Cells (10^6^) were washed twice in cold PBS, lysed by RIPA sample buffer with proteinase and phosphatase inhibitor (Keygen, Nanjing, China), placed in an iced bath for 30 min, and centrifugated at 10625 x g for 10 min at 4°C. The supernatants were obtained for determining the protein concentrations. Samples were boiled with protein buffer solution including 5% β-mercaptoethanol for 5 min at 100°C, then centrifugated at 10625 x g for 1 min at room temperature. Proteins were separated on 10% or 12% SDS-polyacrylamide gel electrophoresis (SDS-PAGE) and transferred onto PVDF membranes (0.2 *μ*M, Mllipore, São Paulo, SP, Brazil). Non-specific-binding sites were blocked with 5% non-fat dry milk dissolved in TBS (10 mM Tris-HCl,pH 7.6, 137 mM NaCl) with 0.1% Tween 20 (TTBS) for 2 h at room temperature followed by incubation with primary antibody at 4°C overnight. The membranes were then washed 3 times in TTBS and incubated for 2 h at room temperature with secondary horseradish peroxidase (HRP)-conjugated donkey anti-rabbit antibody or HRP-conjugated sheep anti-mouse antibody diluted 1:5000 in TTBS with 5% non-fat milk. Proteins were visualized by enhanced chemiluminescent (ECL) plus (Amersham Biosciences, Piscataway, NJ). All experiments were carried out independently at least 3 times. The level of the GAPDH protein was used as a control of the amount of protein loaded into each lane.

### Statistical analysis

All assays were performed in triplicate, and the data were expressed as mean values ±SD. The Student’s t-test was used to compare two groups. Results were considered significant with P-value <0.05.

## Results

**Fig 1:**
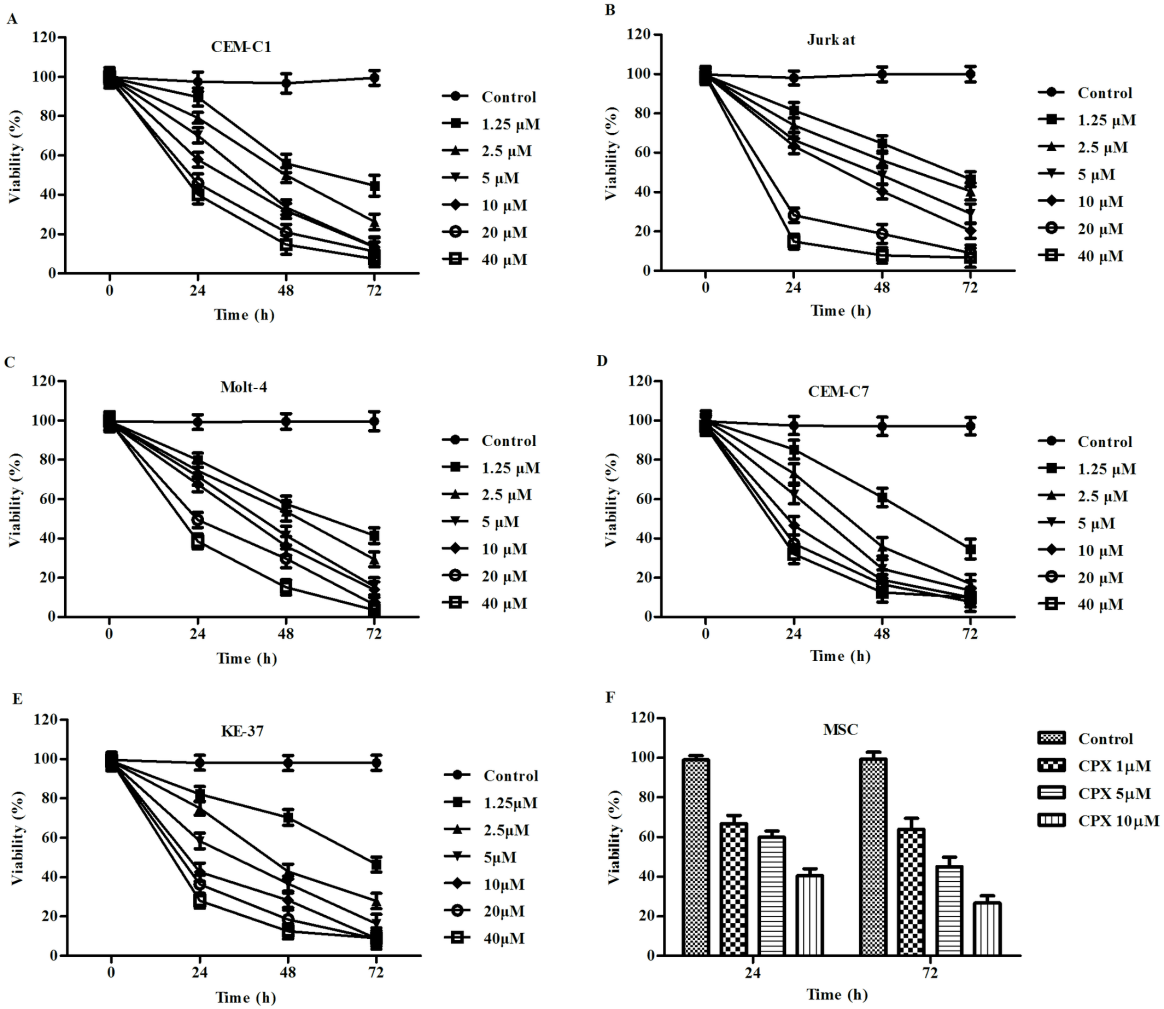
Ciclopirox olamine (CPX) inhibited the growth of the T-ALL cells. (A) to (E) Five T-ALL cell lines (CEM-C1, CEM-C7, Molt-4, Jurkat, and KE-37) were incubated with CPX in different concentrations and for different time courses. The proliferation rate of the cells was evaluated by MTT assay. For each assay, values of triple experiments were shown as mean plus or minus SD. (F) The non-malignant mesenchymal stem cells (MSCs) derived from the bone marrow was also included to test the cytotoxity of CPX on normal cells. In all of the 5 T-ALL cell lines, with CPX concentrations ranging from 1.25 to 40 *μ*M, the percentage of viable cells were all significantly lower as compared to their control groups after 24, 48, and 72 h incubation, P<0.05.

CPX inhibited the growth of T-ALL cells. To evaluate the potential antileukemia effect of CPX on GC-resistant T-ALL cells, we selected a panel of five T-ALL cell lines, three GC-resistant cell lines, CEM-C1, Molt-4, and Jurkat, and two GC-sensitive cell lines, CEM-C7 and KE-37, which were used as controls. Five cell lines were incubated for 72 h with different concentrations of CPX ranging from 1.25 to 40 *μ*M. CPX inhibited the growth of all five T-ALL cell lines in a time and dosage dependent manner (Fig 1A to 1E). CPX at the concentration of 10 *μ*M showed a marked inhibitory effect on all of the 5 tumor cell lines, but had lesser cytotoxicity on the non-malignant mesenchymal stem cells derived from bone marrow (Fig 1F). Since animal model studies indicated that the serum concentrations of 10*μ*M were achievable after repeated administration of CPX to rats and dogs and were not toxic [16], we choose this concentration for further study. At this concentration, the percentage of viable tumor cells was much lower as compared with their control groups after 24, 48, and 72 h treatment, P<0.05 (Table 1). These data suggest that CPX displays a growth inhibitory effect on T-ALL cells, no matter whether they are GC sensitive or resistant.

**Table 1:** Percentage of viable cells in the presence of 10 *μ*M CPX ($\bar{X}\;\pm \;SD$).

cell	0 h	24 h	48 h	72 h
CEM-C7	97.73±2.22	46.54±3.34^a^	19.09±1.89^a^	9.99±1.79^a^
CEM-C1	99.00±0.59	54.57±9.25^a^	28.49±5.21^a^	12.31±0.87^a^
Jurkat	97.51±3.04	64.11±3.50^a^	40.36±5.50^a^	14.33±2.22^a^
Molt-4	97.37±2.29	65.58±2.50^a^	30.09±5.03^a^	13.27±0.26^a^
KE-37	98.20±1.22	42.54±0.38^a^	28.23±2.36^a^	8.89±1.63^a^
MSC	97.92±0.49	40.60±1.43^a^	34.00±1.23^a^	26.70±2.54^a^

^a^ P<0.05 compared with control.

**Fig 2:**
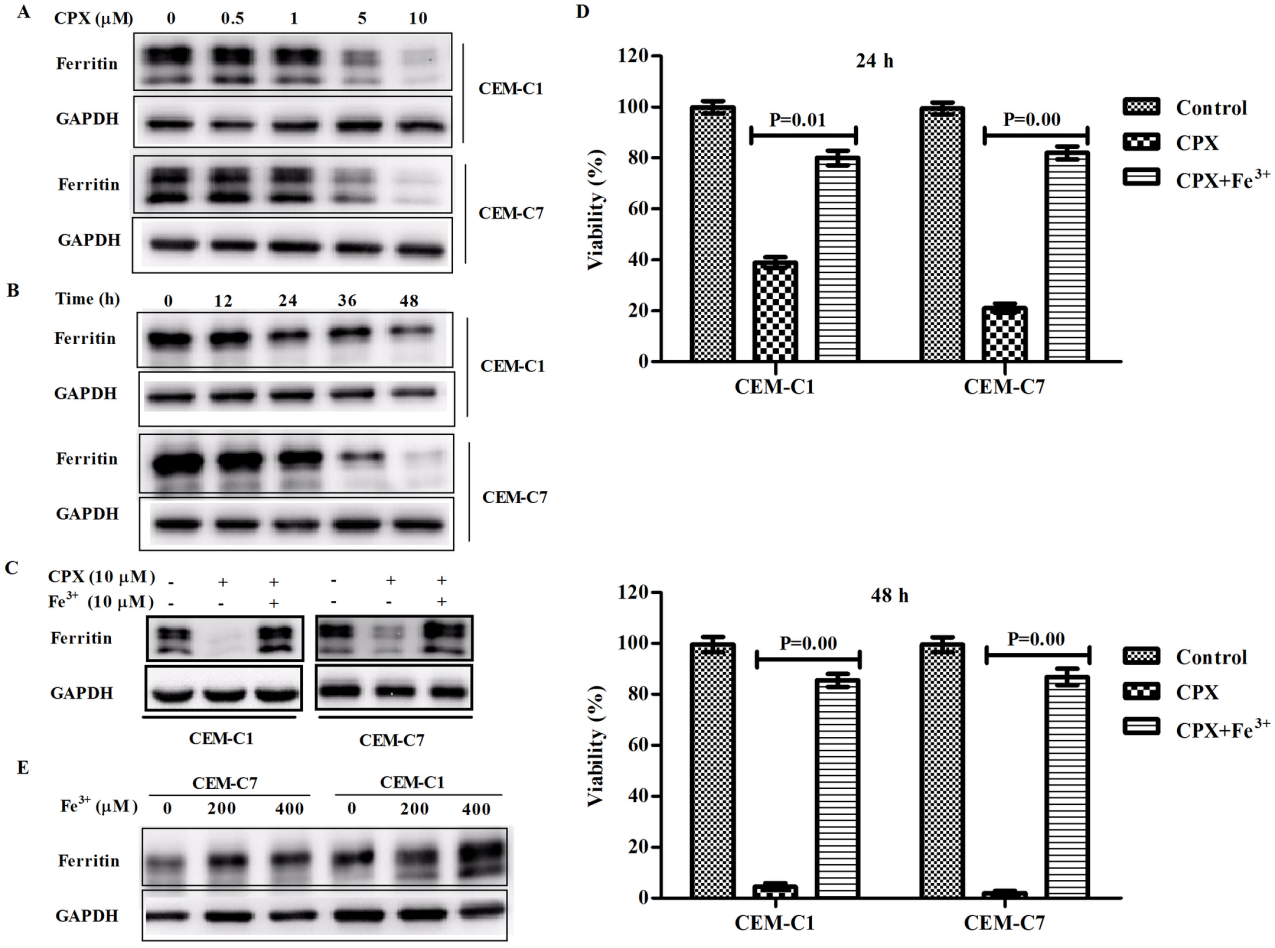
CPX’s inhibitory effect was closely correlated with downregulation of intracellular ferritin expression. (A) Western blot analysis indicated that CPX inhibited the intracellular ferritin expression in both GC-sensitive CEM-C7 and GC-resistant CEM-C1 cells in a dose- dependent manner. (B) At the concentration of 10 *μ*M, CPX inhibited the intracellular ferritin expression in a time-dependent manner. (C) Addition of Fe^3+^ (10 *μ*M) could markedly induce the expression level of intracellular ferritin and blocked the inhibitory effect of CPX on ferritin expression in both GC-resistant CEM-C1 cells and GC-sensitive CEM-C7 cells. (D) Pre-treatment of CEM-C1 cells with Fe^3+^ (10 *μ*M) could totally abrogate the inhibitory effect of CPX on the growth of CEM-C1 and -C7 cells. (E) GC-resistant CEM-C1 cells had a higher capacity of upregulation of ferritin than GC-resistant CEM-C7 cells.

CPX’s inhibitory effect was closely correlated with downregulation of intracellular ferritin expression. CPX is a well-known iron chelator which can bind tightly to intracellular iron molecules to block the ions’ ability to catalyze redox reactions. To see whether CPX has an effect on ferritin expression in the cells, we did Western blot analysis to test the intracellular ferritin levels in the leukemia cells. CPX downregulated the expression of intracellular ferritin in a time- and concentration-dependent manner in GC-sensitive CEM-C7 cells as well as in GC-resistant CEM-C1 cells (Fig 2A and 2B). Pre-treatment of CEM-C1 cells with Fe^3+^ could markedly induce the expression level of intracellular ferritin (Fig 2C) and totally blocked the inhibitory effect of CPX on both CEM-C1 and CEM-C7 cells (Fig 2D). The similar results were also found in the other two GC-resistant cell lines of Jurkat and Molt-4 (Data not shown). Interestingly, although we did not see a difference in ferritin expressions in GC-sensitive and -resistant cells, we found that GC-resistant CEM-C1 cells did have a higher capacity of upregulation of ferritin than GC-sensitive CEM-C7 cells (Fig 2E). These findings suggest that CPX’s antileukemia effect is associated with downregulation of intracellular ferritin expression.

**Fig 3:**
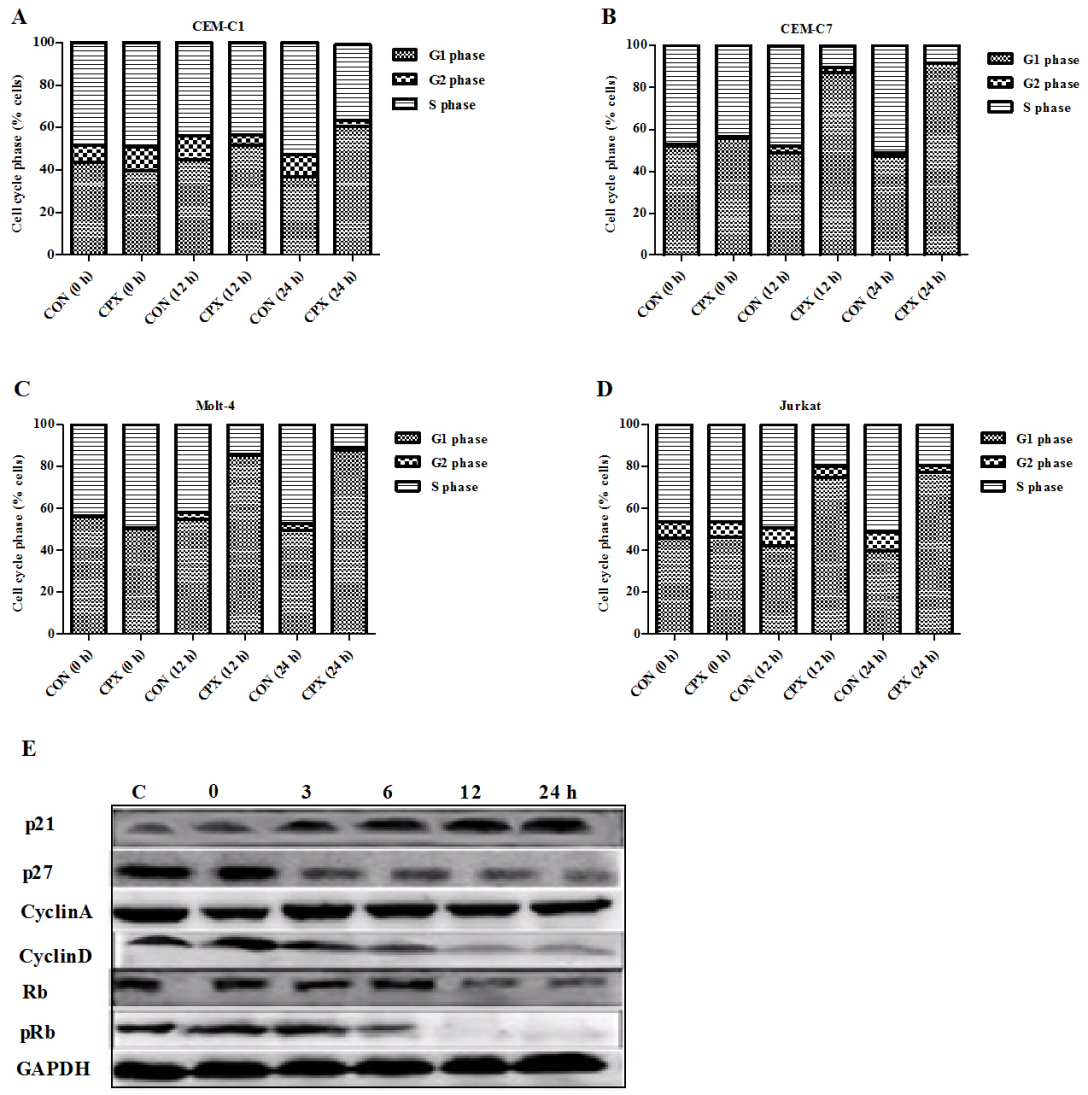
CPX arrested T-ALL cells in G1 phase of the cell cycle. (A) to (D) Flow cytometric analysis showed that CPX (10 *μ*M) clearly induced G1 cell cycle arrest in all the 4 cell lines of T-ALL, especially after 12 h treatment. (E) Western blot analysis indicated that CPX (10 *μ*M) markedly induced upregulation of CDK inhibitor of p21, but inhibited CDK inhibitor of p27, cyclin D, retinoblastoma protein (Rb), and phosphorylated Rb (pRb) in CEM-C1 cells.

CPX arrested T-ALL cells in G1 phase of the cell cycle. Flow cytometric analysis (FACS) showed that CPX clearly induced G1 cell cycle arrest in GC-sensitive CEM-C7 and the other three GC-resistant cell lines of T-ALL. After incubation with CPX at 10 *μ*M for 12 and 24 h, G1-phase cells were increased from originally 39.6 to 50.9 and 60.8% respectively in CEM-C1 cells (Fig 3A), from originally 55.6 to 87.2 and 91.6% in CEM-C7 cells (Fig 3B), from originally 50.7 to 85.3 and 89.0% in Molt-4 cells (Fig 3C), and from originally 46.2 to 72.7 and 77.1% in Jurkat cells (Fig 3D). Compared with their controls, the differences were significant in all of the four cells (P < 0.05). This increase in G1/G0 cell population was accompanied with a concomitant decrease of cell numbers in S phase and G2/M phase of the cell cycle.

Cell-cycle progression is regulated by a series of cyclins and cyclin-dependent kinases (CDKs). To evaluate the molecular basis underlying cell cycle arrest, we investigated the expression of cell cycle regulatory proteins. We chose to test the expressions of cyclin A and D, and CDK inhibitors, p21 and p27. As shown in Fig 3E, CPX markedly induced upregulation of CDK inhibitor of p21, but slightly inhibited p27 expression. It also inhibited cyclin D, but had no effect on the expression of cyclin A. The retinoblastoma protein, Rb, is a tumor suppressor protein that is dysfunctional in several major cancers. Rb restricts the cell's ability to replicate DNA by preventing its progression from the G1 to S phase of the cell division cycle. When the cell is ready to divide, Rb is phosphorylated, becomes inactive, and allows cell cycle progression. CPX inhibited both Rb expression and its phosphorylation, especially the latter.

**Fig 4:**
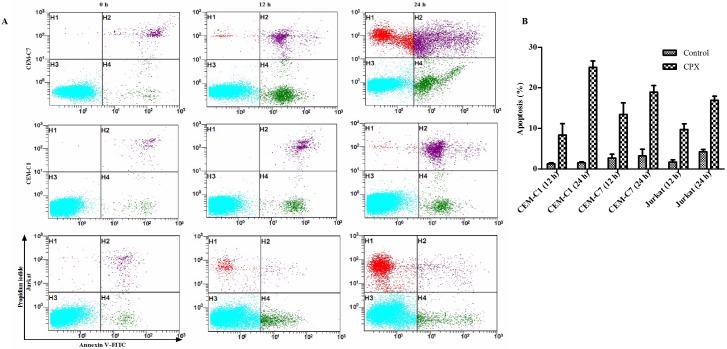
CPX induced apoptosis in T-ALL cells. (A) and (B) Flow cytometric analysis showed an increased rate of early stage of apoptosis (Annexin V-FITC+/PI-, H4) after 12 h treatment of CPX (10 *μ*M) and both rates of the early and late phase of apoptotic cell death (Annexin V-FITC+/PI+, H2) were increased after 24 h treatment in GC resistant cells of CEM-C1 and Jurkat, as well as GC-sensitive CEM-C7 cells.

**Fig 5:**
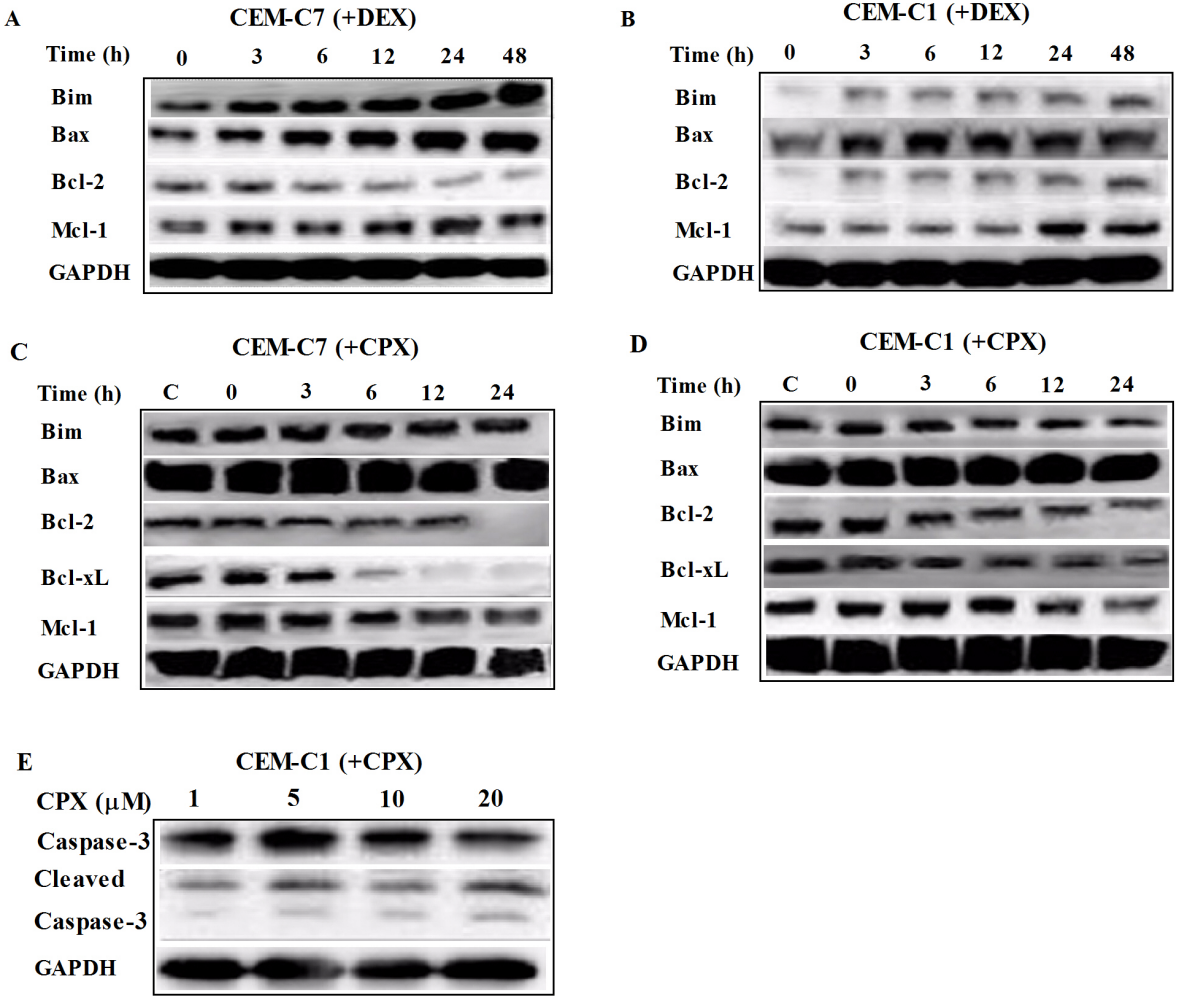
CPX induced apoptosis in T-ALL cells by downregulation of anti-apoptosis proteins. (A) and (B) Western blot analysis indicated that DEX induced the expression of pro-apoptosis proteins of Bim and Bax only in GC-sensitive cells of CEM-C7 and no obvious inhibition of anti-apoptosis proteins in both GC-sensitive and -resistant cells. (C) and (D) However, CPX at the concentration of 10 *μ*M inhibited the expressions of anti-apoptosis proteins, Bcl-2, Mcl-1, and Bcl-xL instead of inducing the expressions of pro-apoptotic proteins of Bim and Bax in both GC-sensitive CEM-C7 cells as well as in GC-resistant CEM-C1 cells. (E) CPX, at the concentration ranging from 1 to 20 *μ*M, activated Caspase-3 by induction of its two processed forms in CEM-C1 cells.

CPX induced apoptosis in T-ALL cells. Annexin V-FITC and PI staining were used to determine the early and late stage of apoptosis. After 12 h treatment with CPX at 10 *μ*M, there was an increased rate of early stages of apoptosis (Annexin V-FITC+/PI-) in all three GC-resistant cells as well as the GC-sensitive CEM-C7 cells. After 24 h treatment, both the early and late phases of apoptotic cell death (Annexin V-FITC+/PI+) were increased (Fig 4A, 4B and Table 2). Taken together, these results suggested that CPX induced apoptosis in T-ALL cells. To understand the mechanism by which CPX induced apoptosis of the T-ALL cells, we then examined the expressions of apoptosis related proteins by Western blot analysis. Compared with GC-resistant CEM-C1 cells, DEX exerted its antileukemia effect in GC-sensitive CEM-C7 cells mainly by inducing pro-apoptosis proteins of Bim and Bax instead of inhibiting anti-apoptosis proteins such as of Bcl-2 and Mcl-1 (Fig 5A and 5B). However, after CPX treatment, there was no obvious upregulation of pro-apoptotic proteins of Bim and Bax, but there was a clear decrease in the expressions of the anti-apoptosis proteins of Bcl-2, Mcl-1, and Bcl-xL in both GC-resistant CEM-C1 and GC-sensitive CEM-C7 cells (Fig 5C and 5D), which showed no changes with DEX treatment. Caspase-3 is activated in both extrinsic and intrinsic apoptotic pathways. Western blot analysis in CEM-C1 cells indicated that CPX activated Caspase-3 pathway by induction of its two processed forms, although there was not a clear-cut time- and dosage-dependent manner (Fig 5E). Similar findings were seen in other T-ALL cell lines. These data support that CPX induces apoptosis in T-ALL cells by downregulation of anti-apoptosis proteins instead of upregulation of pro-apoptosis proteins.

**Table 2:** Percentage of apoptosis in the presence of 10 *μ*M CPX ($\bar{X}\;\pm \;SD$).

	H1	H2	H3	H4
CEM-C7 (0 h)	0.15±0.18	1.45±1.02	97.28±2.39	1.25±0.63
CEM-C7 (12 h)	0.48±0.28	3.51±0.46	86.08±2.41	9.94±2.86
CEM-C7 (24h)	8.46±3.57	4.00±1.59	72.62±2.30	14.97±5.28
CEM-C1 (0 h)	0.06±0.04	0.41±0.26	98.63±0.44	0.86±0.21
CEM-C1 (12 h)	0.01±0.02	2.23±0.40	90.23±0.33	6.13±2.84
CEM-C1 (24 h)	5.30±3.11	6.87±1.60	77.82±5.47	18.16±8.40
Jurkat (0 h)	0.33±0.06	1.93±1.53	96.03±0.29	1.73±0.15
Jurkat (12 h)	0.87±0.47	2.30±0.36	89.40±1.31	7.43±1.40
Jurkat (24 h)	12.23±1.66	5.93±0.85	74.33±3.85	11.00±1.04

Notes: H1, PI (+); H2, PI (+)/Annexin-V (+); H3, PI (-)/Annexin-V (-); H4, Annexin-V (+).

**Fig 6:**
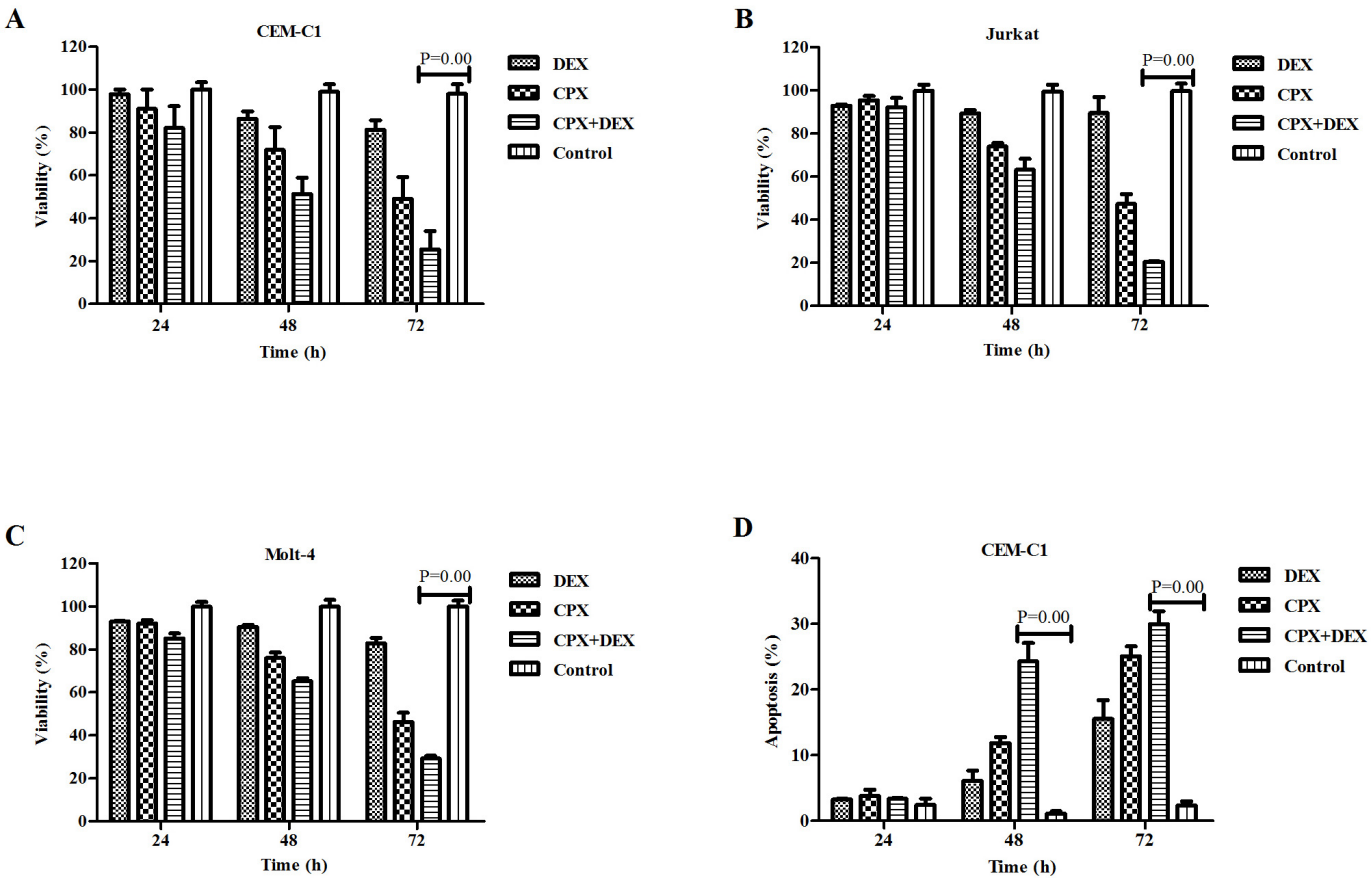
CPX has a strong synergistic antileukemia effect with dexamethasone (DEX) in GC-resistant T-ALL cells. (A) to (C) When combined with DEX (1 *μ*M), CPX demonstrated a potent growth inhibition of the three GC-resistant cells even at a concentration as low as 1 *μ*M, especially after 72 h exposure. (D) Flow cytometric analysis showed that CPX (1 *μ*M) had a synergistic effect on inducing apotosis after 48 h treatment with DEX (1 *μ*M) in CEM-C1 cells. D+C; DEX+CPX.

**Fig 7:**
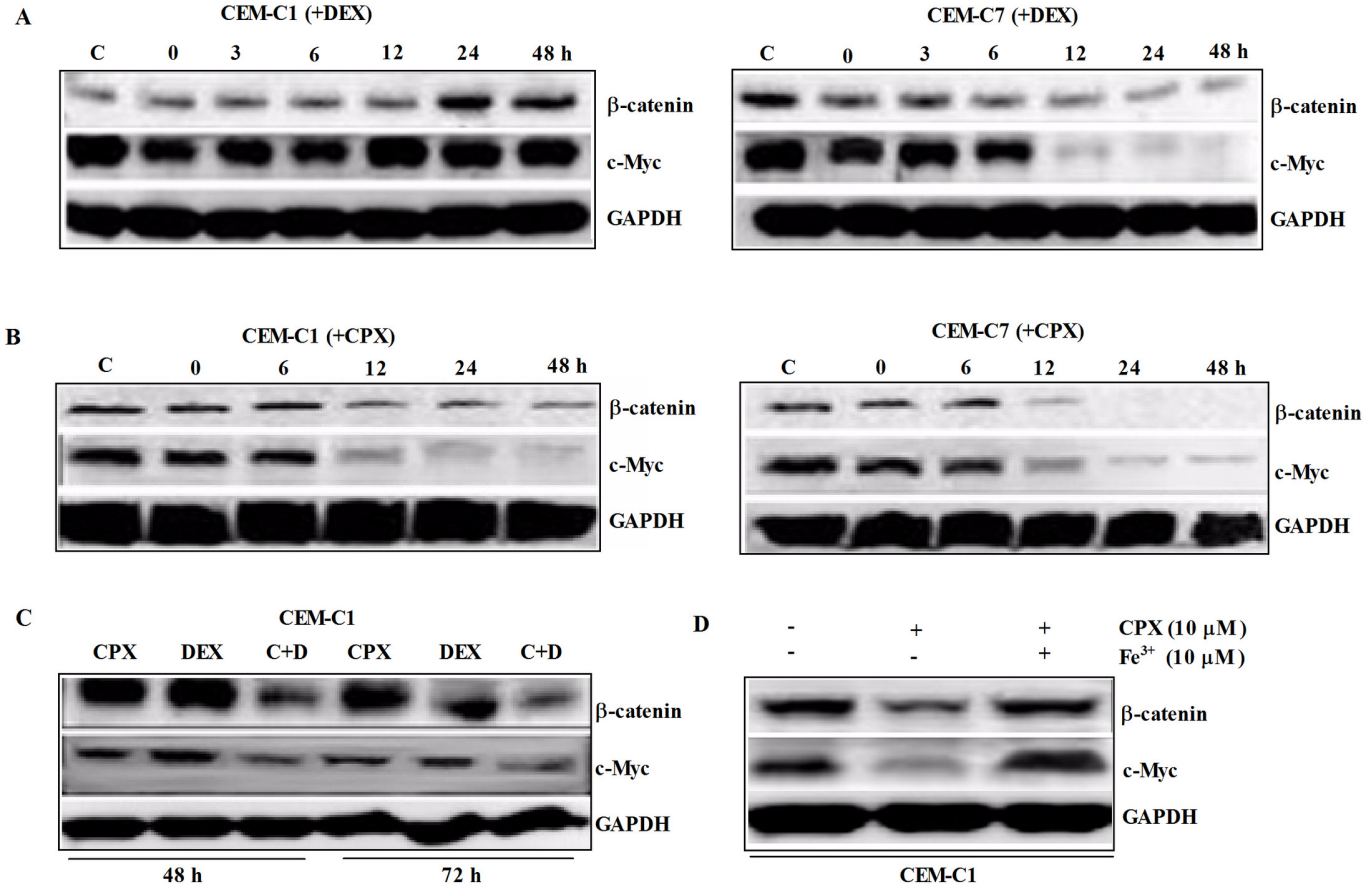
A marked synergistic inhibition on the expressions of both c-Myc and β-catenin was induced by CPX when used in combination with DEX. (A) DEX (1 *μ*M) inhibited c-Myc expression only in GC-sensitive CEM-C7 cells. (B) 10 *μ*M CPX inhibited c-Myc and β-catenin expressions in both GC-sensitive CEM-C7 cells and GC-resistant CEM-C1 cells. (C) 1 *μ*M CPX had no obvious inhibition on the expressions of c-Myc and β-catenin, but when it was used with DEX (1 *μ*M), there was a marked synergistic inhibition on the expressions of both c-Myc and β-catenin. (D) Pre-treatment of the tumor cells with Fe^3+^ blocked the inhibitory effect of CPX on the expressions of β-catenin and c-Myc.

CPX demonstrated a strong synergistic antileukemia effect with DEX in T-ALL cells. CPX inhibited the growth of both GC-sensitive and GC-resistant T-ALL cells in a time- and concentration-dependent manner. Moreover, CPX had a strong synergistic antileukemia effect with DEX in GC-resistant cells. When it was used in combination with DEX, even at a concentration as low as 1 *μ*M, it demonstrated a potent growth inhibition in all 3 GC-resistant cells, especially after 72 h of exposure (Fig 6A–6C). Cytometric analysis demonstrated that CPX and DEX had an increased synergistic effect on inducing apoptosis after 24 h of co-treatment of the CEM-C1 cells (Fig 6D). DEX could downregulate the expressions of β-catenin and c-Myc only on GC sensitive CEM-C7 cells (Fig 7A), whereas 10 *μ*M CPX could downregulate these two proteins in both GC-sensitive CEM-C7 cells and GC-resistant CEM-C1cells (Fig 7B). A low concentration of CPX at 1 *μ*M had no effect on inhibition of the protein expressions in CEM-C1 cells, but when it was used together with DEX, there was a marked synergistic inhibition on the expression of both β-catenin and c-Myc in CEM-C1 cells (Fig 7C). However, pre-treatment of the tumor cells with Fe^3+^ could block the inhibitory effect of CPX on the expressions of β-catenin and c-Myc (Fig 7D). These data suggest that the synergistic antileukemia effect of CPX and DEX might be mediated by inhibition of the β-catenin-c-Myc signaling pathway.

## Discussion

T-ALLs are aggressive proliferations of transformed T-cell progenitors, with overall survival rates of 70% in children and 40% in adults [10, 11]. Patients with T-ALL have historically been classified as a risk group and experience a worse prognosis [12, 20–22]. Although intensification of chemotherapeutic schedules greatly improved its prognosis in the past 10 years, about 15% of pediatric and 40% of adult T-ALL patients still relapse due to acquired therapy resistance within the first 2 years following diagnosis and present very dismal survival perspectives [2, 3, 11]. GCs are one of the most utilized and the most effective therapies in treating T-ALLs, however, patients often develop resistance to GCs, rendering these therapies ineffective [23–25]. Also, primary GC resistance which confers a much higher risk of relapse is particularly prevalent in T-ALL compared to B-precursor leukemias [26]. Therefore, to improve the prognosis of T-ALL, it is essential to define the mechanism of GC resistance, identify pathways leading to it and finally find new therapeutic reagents to overcome it.

CPX is currently approved for the topical treatment of cutaneous fungal infections and available in a variety of topical formulations including gels, creams, lacquers, and shampoos [19]. Recently, preclinical studies showed that systemic administration of CPX as an antitumor agent in advanced hematologic malignancies was well tolerated and displayed biological activity [17]. Although the exact mechanism of ciclopirox is unclear, its antitumor activity has been attributed to iron chelation [16]. By chelating intracellular iron, CPX inhibits iron-dependent enzymes such as ribonucleotide reductase [27, 28] and disrupts iron-dependent cellular processes such as Wnt signaling [29, 30]. In this study, we found that CPX had antileukemia effects on both GC-sensitive and GC-resistant T-ALL cells, and this effect was closely correlated with downregulation of intracellular ferritin since addition of FeCl_3_ could markedly induce ferritin expression and totally blocked its antileukemia effect.

Ferritin is a ubiquitous intracellular protein that stores iron, which is an essential element for mammalian cell growth and proliferation. It is a globular protein complex consisting of 24 protein subunits composed of two subunit types, termed H and L chain [31]. The ferritin H subunit has a potent ferroxidase activity that catalyses the oxidation of ferrous iron, whereas ferritin L plays a role in iron nucleation and protein stability [32]. Ferritin is found in most tissues as a cytosolic protein, but small amounts are secreted into the serum where it functions as an iron carrier. In humans, it acts as a buffer against iron deficiency and iron overload. However, more evidence suggest that ferritin is differentially over-expressed in tissues from multiple malignancies, including hepatocellular carcinoma, Hodgkin's lymphoma, breast cancer, pancreatic cancer, and acute leukemias [33]. Also there are data suggesting that high ferritin expression can enhance cell growth and improve resistance to oxidative stress by inhibiting the accumulation of radical oxygen species (ROS) aftertreatment with oxidative insults in metastatic melanoma and breast cancer cells by interfering with their cellular antioxidant system, and its downregulation could disrupt the supportive tumor microenvironment, kill cancer cells, and increase sensitivity to chemotherapy, suggesting that ferritin could be an attractive target for cancer therapy [34]. In our study, we did not see a difference in ferritin expressions in GC-sensitive and -resistant cells, but GC resistant CEM-C1 cells did have a higher capacity of upregulation of ferritin than GC-sensitive CEM-C7 cells. CPX demonstrated an equally inhibitive effect on ferritin expression in both GC-sensitive and GC- resistance T-ALL cells, which would explain its effectiveness in both GC-sensitive and GC-resistance cells.

Chromosomal aberrations leading to abnormal fusion proteins are not commonly found in T-ALL, however, aberrant regulations of signaling pathways that control normal T-cell development in the thymus are important for T-ALL leukomogenesis [35]. Constitutive activation of NOTCH and β-catenin-mediated WNT signaling pathways are two of the most important and common alternations found in T-ALL [35, 36], and c-Myc is the direct transcriptional target of both pathways. In this study, we showed that DEX could inhibit both c-Myc and β-catenin expressions in GC- sensitive CEM-C7 cells, but not in GC-resistant CEM-C1 cells. High concentration of CPX at 10 *μ*M alone could inhibit β-catenin and c-Myc expressions in both GC- sensitive CEM-C7 and GC-resistant CEM-C1 cells. Importantly, CPX, even at a lower concentration of 1 *μ*M, had a strong synergistic effect with DEX in inhibiting the expressions of β-catenin and c-Myc, especially the latter. These data would suggest that CPX’s antileukemia effect in GC-resistant cells might be mediated by directly targeting iron-dependent (WNT)/β-catenin signaling pathway and indirectly NOTCH pathway through inhibiting c-Myc expression.

We also demonstrated that CPX could induce cell cycle arrest at G1 phase in all of the four T-ALL cell lines. CPX downregulated the expressions of cyclin D, Rb, and phosphorylated Rb (pRb) but had no effect on cyclin A. Mechanically CPX induced cell cycle arrest by upregulation of cyclin-dependent kinase (CDK) inhibitor of p21, which can bind and inhibit the cyclin D, E, and A-dependent kinases (CDK2 and CDK4), but surprisingly CPX inhibited CDK inhibitor of p27 which can inhibit the activity of cyclin E or A containing CDK2. These findings are consistent with the results of a similar study in another human solid tumor cell line, rhabdomyosarcoma (Rh30) cells [23].

CPX could also induce apoptosis in GC-resistant T-ALL cells. Unlike PI3K/AKT/mTOR pathway inhibitors which can re-sensitize T-ALL cells to GC treatment by upregulation of pro-apoptotic proteins of Bim and Bax [37], CPX mainly downregulated the expression of anti-apoptosis proteins of Bcl-2, Mcl-1, and Bcl-xL.

In conclusion, CPX displays antileukemia effect on both GC-sensitive and GC- resistant T-ALL cells, and this is closely correlated with downregulation of intracellular ferritin expression and inhibition of the β-catein-c-Myc signaling pathway. CPX shows a strong synergistic antileukemia effect with GC in GC-resistant T-ALL cells. These data suggest that modulation of the intracellular ferritin expression in leukemia cells might be an effective method in the treatment of ALL, and integration of CPX into the current GC-containing ALL protocols could lead to improvement of treatment options in ALL, especially in GC-resistant ALL.

## References

[1] Pui CH, Pei D, Campana D, Cheng C, Sandlund JT, Bowman WP, et al A revised definition for cure of childhood acute lymphoblastic leukemia. Leukemia. 2014; 28: 2336–43. 10.1038/leu.2014.142 24781017 PMC4214904

[2] Cooper SL, Brown PA. Treatment of pediatric acute lymphoblastic leukemia. Pediatr Clin North Am. 2015; 62: 61–73. 10.1016/j.pcl.2014.09.006 25435112 PMC4366417

[3] Hunger SP, Mullighan CG. Redefining ALL classification: toward detecting high-risk ALL and implementing precision medicine. Blood. 2015; 125: 3977–87. 10.1182/blood-2015-02-580043 25999453 PMC4481590

[4] Gaynon PS, Lustig RH. The use of glucocorticoids in acute lymphoblastic leukemia of childhood: molecular, cellular, and clinical considerations. Pediatr Hematol Oncol. 1995; 17: 1–12. 10.1097/00043426-199502000-00001 7743230

[5] Greenstein S, Ghias K, Krett NL, Rosen ST. Mechanisms of glucocorticoidmediated apoptosis in hematological malignancies. Clin Cancer Res. 2002; 8: 1681–94. 12060604

[6] Bhadri VA, Trahair TN, Lock RB. Glucocorticoid resistance in paediatric acute lymphoblastic leukaemia. J Paediatr Child Health. 2012; 48: 634–40. 10.1111/j.1440-1754.2011.02212.x 22050419

[7] Marino S, Verzegnassi F, Tamaro P, Stocco G, Bartoli F, Decorti G, et al Response to glucocorticoids and toxicity in childhood acute lymphoblastic leukemia: role of polymorphisms of genes involved in glucocorticoid response. Pediatr Blood Cancer. 2009; 53: 984–91. 10.1002/pbc.22163 19621425

[8] Schrappe M. Evolution of BFM trials for childhood ALL. Ann Hematol. 2004; 83 (Suppl 1): S121–S123. 15124702 10.1007/s00277-004-0850-2

[9] Kaspers GJ, Pieters R, Klumper E, De Waal FC, Veerman AJ. Glucocorticoid resistance in childhood leukemia. Leuk Lymphoma. 1994; 13: 187–201. 8049644 10.3109/10428199409056282

[10] van Grotel M, Meijerink JP, van Wering ER, Langerak AW, Beverloo HB, Buijs-Gladdines JG, et al Prognostic significance of molecular-cytogenetic abnormalities in pediatric T-ALL is not explained by immunophenotypic differences. Leukemia. 2008; 22: 124–31. 17928886 10.1038/sj.leu.2404957

[11] Durinck K, Goossens S, Peirs S, Wallaert A, Van Loocke W, Matthijssens F, et al Novel biological insights in T-cell acute lymphoblastic leukemia. Exp Hematol. 2015; 43: 625–39. 10.1016/j.exphem.2015.05.017 26123366

[12] Uckun FM, Sensel MG, Sun L, et al Biology and treatment of childhood T-lineage acute lymphoblastic leukemia. Blood. 1998; 91: 735–46. 9446631

[13] Sehgal VN. Ciclopirox: a new topical pyrodonium antimycotic agent. A double-blind study in superficial dermatomycoses. Br J Dermatol. 1976; 95: 83–8. 10.1111/j.1365-2133.1976.tb15537.x 782504

[14] Täuber A, Müller-Goymann CC. In vitro model of infected stratum corneum for the efficacy evaluation of poloxamer 407-based formulations of ciclopirox olamine against Trichophyton rubrum as well as differential scanning calorimetry and stability studies. Int J Pharm. 2015; 494: 304–11. 10.1016/j.ijpharm.2015.08.023 26276254

[15] Zhou H, Shen T, Luo Y, Liu L, Chen W, Xu B, et al The antitumor activity of the fungicide ciclopirox. Int J Cancer. 2010; 127: 2467–77. 10.1002/ijc.25255 20225320 PMC2888914

[16] Eberhard Y, McDermott SP, Wang X, Gronda M, Venugopal A, Wood TE, et al Chelation of intracellular iron with the antifungal agent ciclopirox olamine induces cell death in leukemia and myeloma cells. Blood. 2009; 114: 3064–73. 10.1182/blood-2009-03-209965 19589922

[17] Minden MD, Hogge DE, Weir SJ, Kasper J, Webster DA, Patton L, et al Oral ciclopirox olamine displays biological activity in a phase I study in patients with advanced hematologic malignancies. Am J Hematol. 2014; 89: 363–8. 10.1002/ajh.23640 24273151

[18] Sen S, Hassane DC, Corbett C, Becker MW, Jordan CT, Guzman ML. Novel mTOR inhibitory activity of ciclopirox enhances parthenolide antileukemia activity. Exp Hematol. 2013; 41: 799–807. 10.1016/j.exphem.2013.04.012 23660068 PMC3809917

[19] Weir SJ, Patton L, Castle K, Rajewski L, Kasper J, Schimmer AD. The repositioning of the anti-fungal agent ciclopirox olamine as a novel therapeutic agent for the treatment of haematologic malignancy. J Clin Pharm Ther. 2011; 36: 128–34. 10.1111/j.1365-2710.2010.01172.x 21366640

[20] Möricke A, Reiter A, Zimmermann M, Gadner H, Stanulla M, Dördelmann M, et al Risk-adjusted therapy of acute lymphoblastic leukemia can decrease treatment burden and improve survival: treatment results of 2169 unselected pediatric and adolescent patients enrolled in the trial ALL-BFM 95. Blood. 2008; 111: 4477–89. 10.1182/blood-2007-09-112920 18285545

[21] Goldberg JM, Silverman LB, Levy DE, Dalton VK, Gelber RD, Lehmann L, et al Childhood T-cell acute lymphoblastic leukemia: the Dana-Farber Cancer Institute acute lymphoblastic leukemia consortium experience. J Clin Oncol. 2003; 21: 3616–22. 14512392 10.1200/JCO.2003.10.116

[22] You MJ, Medeiros LJ, His ED. T-Lymphoblastic Leukemia/Lymphoma. Am J Clin Pathol. 2015; 144: 411–22. 10.1309/AJCPMF03LVSBLHPJ 26276771

[23] Reiter A, Schrappe M, Ludwig WD, Hiddemann W, Sauter S, Henze G, et al Chemotherapy in 998 unselected childhood acute lymphoblastic leukemia patients: results and conclusions of the multicenter trial ALL-BFM 86. Blood. 1994; 84: 3122–33. 7949185

[24] Griffin TC, Shuster JJ, Buchanan GR, Murphy SB, Camitta BM, Amylon MD. Slow disappearance of peripheral blood blasts is an adverse prognostic factor in childhood T cell acute lymphoblastic leukemia: a Pediatric Oncology Group study. Leukemia. 2000; 14: 792–5. 10803508 10.1038/sj.leu.2401768

[25] Aricò M, Basso G, Mandelli F, Rizzari C, Colella R, Barisone E, et al Good steroid response in vivo predicts a favorable outcome in children with T-cell acute lymphoblastic leukemia. The Associazione Italiana Ematologia Oncologia Pediatrica (AIEOP). Cancer. 1995; 75: 1684–93. 8826928 10.1002/1097-0142(19950401)75:7<1684::aid-cncr2820750720>3.0.co;2-2

[26] Bhadri VA, Trahair TN, Lock RB. Glucocorticoid resistance in paediatric acute lymphoblastic leukaemia. J Paediatr Child Health. 2012; 48: 634–40. 10.1111/j.1440-1754.2011.02212.x 22050419

[27] Niewerth M, Kunze D, Seibold M, Schaller M, Korting HC, Hube B. Ciclopirox olamine treatment affects the expression pattern of Candida albicans genes encoding virulence factors, iron metabolism proteins, and drug resistance factors. Antimicrob Agents Chemother. 2003; 47: 1805–17. 12760852 10.1128/AAC.47.6.1805-1817.2003 PMC155814

[28] Trachootham D, Alexandre J, Huang P. Targeting cancer cells by ROS-mediated mechanisms: a radical therapeutic approach? Nat Rev Drug Discov. 2009; 8: 579–91. 10.1038/nrd2803 19478820

[29] Wall I, Schmidt-Wolf IG. Effect of Wnt inhibitors in pancreatic cancer. Anticancer Res. 2014; 34: 5375–80. 25275031

[30] Schmeel LC, Schmeel FC, Kim Y, Endo T, Lu D, Schmidt-Wolf IG. Targeting the Wnt/beta-catenin pathway in multiple myeloma. Anticancer Res. 2013; 33: 4719–26. 24222106

[31] Orino K, Lehman L, Tsuji Y, Ayaki H, Torti SV, Torti FM. Ferritin and the response to oxidative stress. Biochem J. 2001; 357: 241–7. 11415455 10.1042/0264-6021:3570241 PMC1221947

[32] Rucker P, Torti FM, Torti SV. Role of H and L subunits in mouse ferritin. J Biol Chem. 1996; 271: 33352–7. 8969195 10.1074/jbc.271.52.33352

[33] Ahmed A. Alkhateeb, James R. Connor. The significance of ferritin in cancer: Anti-oxidation, inflammation and tumorigenesis. Biochim Biophys Acta. 2013; 1836: 245–54. 10.1016/j.bbcan.2013.07.002 23891969

[34] Baldi A, Lombardi D, Russo P, Palescandolo E, De Luca A, Santini D, et al Ferritin contributes to melanoma progression by modulating cell growth and sensitivity to oxidative stress. Clin Cancer Res. 2005; 11: 3175–83. 15867210 10.1158/1078-0432.CCR-04-0631

[35] Ng OH, Erbilgin Y, Firtina S, Celkan T, Karakas Z, Aydogan G, et al Deregulated WNT signaling in childhood T-cell acute lymphoblastic leukemia. Blood Cancer J. 2014; 4: e192 10.1038/bcj.2014.12 24632884 PMC3972698

[36] Witkowski MT, Cimmino L, Hu Y, Trimarchi T, Tagoh H, McKenzie MD, et al Activated Notch counteracts Ikaros tumor suppression in mouse and human T-cell acute lymphoblastic leukemia. Leukemia. 2015; 29: 1301–11. 10.1038/leu.2015.27 25655195 PMC4845663

[37] Gu L, Zhou CY, Liu HJ, Gao J, Li Q, Mu DZ, et al Rapamycin sensitizes T-ALL cells to dexamethasone-induced apoptosis. J Exp Clin Cancer Res. 2010; 29: 150 10.1186/1756-9966-29-150 21083937 PMC2998469

